# Successful management of chronic urticaria and food allergies in a pediatric population using integrative traditional Chinese medicine therapy: a case series

**DOI:** 10.1186/s12948-022-00175-y

**Published:** 2022-11-25

**Authors:** Xiaowen Fan, Tory McKnight, Johnathan Neshiwat, Song Park, Danna Chung, Xiu-Min Li

**Affiliations:** 1 Department of Medicine, University of Florida College of Medicine, Gainesville, FL 32608 USA; 2 Department of Pathology, Microbiology and Immunology, New York Medical College, Valhalla, NY 10595 USA; 3 Integrative Health and Acupuncture, Mamaroneck, 10543 USA; 4 Department of Medicine, Icahn School of Medicine at Mount Sinai, New York, NY 10029 USA; 5 Department of Otolaryngology, New York Medical College, Valhalla, NY 10595 USA

**Keywords:** Urticaria, Chronic urticaria, Hives, Food allergy, Food sensitivities, IgE, Traditional Chinese medicine

## Abstract

**Background:**

Food allergy is becoming increasingly common among the pediatric population. Despite strict avoidance of food allergens, a subgroup of sensitive individuals still develops frequent, persistent, and difficult to treat hives (FPDTH) for which there is no curative therapy. Although these cases are rare, these patients are in most need of therapy.

**Case presentations:**

This is a retrospective review of 3 pediatric patients with highly sensitive food allergies who initially presented with hives daily or every other day, or multiple times a day, but achieved marked remission after traditional Chinese medicine (TCM) therapies. Patient 1 (P1) is a 5-year-old who has experienced 140 reactions in his lifetime. Reactions were mostly hives with 4 episodes of anaphylaxis. P1 had used Prednisone 20 times, had an Epinephrine injection 4 times, and had 3 emergency room (ER) visits. Patient 2 (P2) is a 12-year-old who had experienced hives since age 3. Despite daily antihistamine use, P2 had > 730 reactions in his lifetime at the time of presentation including 2 episodes of anaphylaxis. He had been prescribed prednisone 4 times, an Epinephrine injection 2 times, and had 1 ER visit. Patient 3 (P3) is a 20-month-old girl who had experienced > 120 reactions including 1 episode of anaphylaxis. She was on daily desonide and frequently used an antihistamine, yet still had required a course of prednisone once, an Epinephrine injection once, and had 1 ER visit to manage her reaction. After presenting to our clinic, patients received internal and external TCM treatments, including herbal baths and creams (Remedy A-D) as basic remedies to reduce food reactions, including but not limited to frequent hives. Within 7–9 months of TCM treatment, remarkably all patients had complete remission of atopic symptoms. All three patients also experienced an improvement in other conditions including food intolerance, diarrhea, anxiety, eczema, and environmental allergies. After 1 year of treatment, all three patients had reductions in food-specific IgE levels that had been previously elevated, and additionally, P1 and P3, who initially had high total IgE levels, experienced a marked decrease in total IgE levels as well. All three patients continued to introduce foods into their diet that they previously had reactions to, and all 3 patients remain symptom-free.

**Conclusions:**

Three pediatric patients with a known history of multiple food sensitivities and physician-diagnosed food allergies that presented with FPDTH underwent a TCM regimen and experienced dramatic improvement in symptoms and reduction in their IgE levels. This regimen appears to be effective in FPDTH population although a further study in a controlled clinical setting is required.

**Supplementary Information:**

The online version contains supplementary material available at 10.1186/s12948-022-00175-y.

## Background

Food allergic reactions, which affect 5% of adults and 8% of children, involve multiple systems including, integumentary (rash and hives), respiratory (cough and wheezing), and gastrointestinal (diarrhea). In food intolerance, the skin is the most commonly affected target organ; the most common manifestation is acute urticaria [1].

Therefore, patients with food intolerance must oftentimes rely on early recognition, strict avoidance, and timely management of reactions in order to prevent serious consequences [2]. These solutions are inherently imperfect. Further, despite strict avoidance, some children with multiple food allergies will continue to have reactions simply from contact with minute, particulate triggers in the environment. Because there is limited FDA-approved treatment for food allergy, these patients experience frequent hives for which there is no curative therapy. There is limited data available for the rare group of children with a history of multiple food allergies and hypersensitive reactions to minute exposure with chronic persistent symptoms that adversely affect their quality of life.

Chronic urticaria, which may occur concurrently in atopic children with food allergies, is also difficult to manage and with too few treatment options. The first line for managing chronic urticaria is a daily dose of a second-generation antihistamine, however over time, patients become desensitized, and the necessary dose can increase up to fourfold without symptom relief. If symptoms persist despite the maximum dose of antihistamine, then other pharmacologics can be used such as omalizumab, cyclosporine, and/or short courses of corticosteroids [3–5]. However, these too are imperfect treatment options. For example, omalizumab has been used in the management of difficult-to-control asthma, chronic urticaria, refractory atopic dermatitis, and food allergy with some positive results, but it is not disease-modifying, as patient’s symptoms recur after stopping treatment, and therefore in certain populations the benefit in clinical outcome versus cost ratio is low [5–9]. The side-effect profiles of chemotherapeutics (i.e. cyclosporine) and corticosteroids are a well-documented consideration, too.

Traditional Chinese Medicine (TCM) has been studied as an alternative in treating many chronic allergic disorders including chronic urticaria. Previous studies have shown that TCM is safe and effective in treating asthma in several randomized, controlled trials [10–14]. TCM also seems to effectively improve the symptoms and decrease the disease severity in patients with moderate to severe atopic dermatitis [15]. A study also showed that the combination of TCM and antihistamine had better clinical outcomes compared to using antihistamine alone in treating chronic urticaria [16]. There have been published studies, both murine models and of patient populations, which describe the efficacy of TCM in treating food allergies as well [17–19]. Notably, a published case study even describes the prevention of frequent, severe anaphylactic food allergies with TCM therapy [20].

The effects of TCM can be attributed to its regulatory effect on helper T cells, B cells, macrophages, and mast cells, which results in cytokine level changes and anti-inflammatory activity [21]. This is a retrospective case study of three pediatric patients with chronic, recalcitrant hives who achieved dramatic symptom improvement following TCM therapy.

## Methods

This is a retrospective review of 3 pediatric patients with highly sensitive food allergies, who experienced hives daily or every other day, or multiple times a day. The copy of laboratory records was provided by the patient’s legal guardian from their local allergists. Patients received internal and external TCM treatments, including herbal baths and creams (Remedy A-D) as basic remedies, to reduce food reactions, including frequent hives. For all 3 patients, laboratory testing data on liver and kidney function were within the normal range during the entire course of the treatment. Patients were seen at the Ming Qi Natural Health Care Center, and Comprehensive Allergy and Immunology, in conjunction with Integrative Health and Acupuncture PC in New York, NY.

Basic remedies, herbal constituents, and doses are described in Appendix A (Additional File 1). Use of additional herbs is noted in each case discussion as applicable.

### Case 1

Patient 1(P1) is a 5-year-old male, with a history of asthma, virus-induced wheezing, allergic rhinitis, eczema, and food hypersensitivity presented to the authors for control of his atopic disease. He also has a history of fire ant anyphylaxis. He had been diagnosed with an egg allergy at 11 months old after having a major reaction with widespread full body hives**,** nasal congestion, sneezing, runny nose, watery eyes, and severe itching. P1 continued to develop additional food allergies including peanut and milk at 12 months old and the frequency of reactions was increasing. In the two years prior to TCM therapy, P1 had 140 reactions, mostly hives but including 4 episodes of anaphylaxis. He had required prednisone therapy 20 times, an epinephrine injection 4 times, and had 3 emergency room (ER) visits related to his food sensitivity. His use of antihistamines 140 times was consistent with his number of allergic reactions (Table 1). Three months prior to TCM, the frequency of his skin reactions increased further; by the time he presented to the authors, he was experiencing daily hives.

**Table 1:** Frequency of reaction, medication use and ER visits before, during and after TCM therapy

# of Uses or Occurrences	Before TCM			During and after the courses of TCM			
	2 Year/Total	Prior 3 M	Prior 1 M	1–3 M	4–6 M	7–9 M	10–12 M
Patient 1 (5 years old)							
Allergic reactions	140	90	30	0	1	0	0
Epinephrine	4	0	0	0	0	0	0
Diphenhydramine	140	90	30	0	0	0	0
Prednisone	20	1	0	0	0	0	0
ER visits	3	0	0	0	0	0	0
Patient 2 (12 years old)							
Allergic reactions	> 730	> 90	30	90	90	0	0
Epinephrine	2	1	1	0	0	0	0
Diphenhydramine	> 100	> 90	30	1	1	0	0
Prednisone	4	1	0	0	0	0	0
ER visits	1	1	0	0	0	0	0
Patient 3 (20 month old)							
Allergic reactions	> 120	> 90	30	1	0	0	0
Epinephrine	1	0	0	0	0	0	0
Diphenhydramine	> 45	45	15	0	0	0	0
Prednisone	1	0	0	0	0	0	0
ER visits	1	0	0	0	0	0	0

**Fig. 1:**
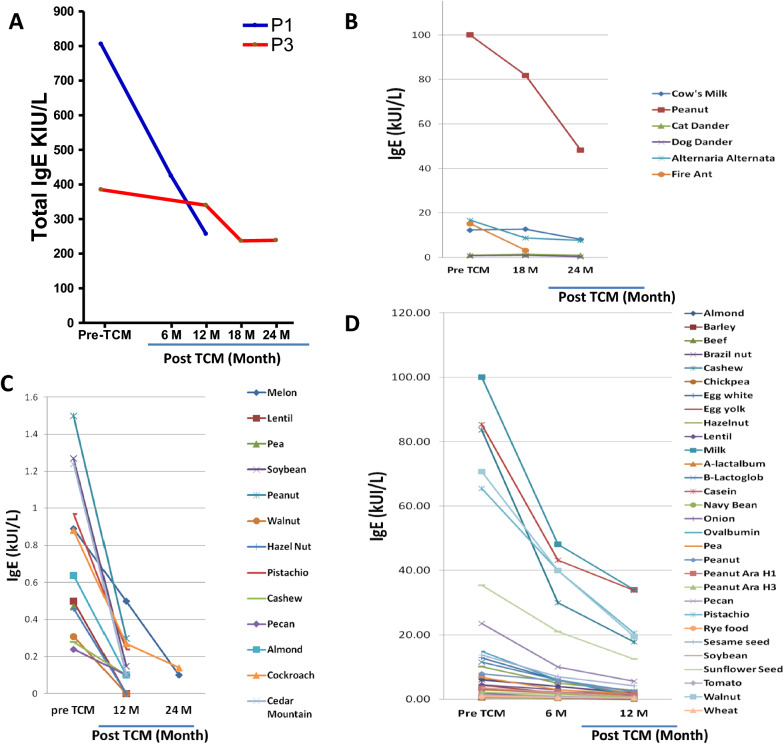
Total and antigen-specific IgE levels. (**A**) Total IgE level changes in patients 1 (blue line) and 3 (red line), respectively. P2 had low total IgE levels that were not included in the figure. Total IgE levels were shown immediately pre-treatment (Pre-TCM), 6, 12,18, and 24 months (M) after treatment started. (**B**) Antigen-specific IgE levels of P1. (**C**) Antigen-specific IgE levels of P2. (**D**) Antigen-specific IgE levels of P3 before and after TCM therapy

P1 received TCM basic remedies A, B, and D plus a supplemental method (Digestion Tea, Remedy E). The goal of reducing hives was achieved within 7–9 months of TCM therapy. During the first 3 months of TCM, the number of episodes of hives declined from daily to none (Table 1). During 4–6 months of TCM, he experienced a single episode of hives, and his asthma, appetite, and eczema improved. At 12 months of TCM, his total and peanut-specific serum IgE levels reduced by half (Fig. 1). At 1.5 years of TCM therapy, he reported no hives whatsoever and was able to safely reintroduce foods, like egg, wheat, and beans which had previously incited reactions.

### Case 2

Patient 2 (P2) is a 12-year-old male with hives since age three and physician-diagnosed food allergies (FA) since age 10 upon experiencing hives, swollen lips, swollen eyes, and a nosebleed after eating a pistachio nut. During subsequent allergy testing at that time, he experienced visible allergic reactions to egg, milk, wheat, peanut, soy, cod, mango, and tomato, but had a minimal elevation of food-specific IgE levels and normal total IgE. At 11 years old, he developed severe reactions following a hazelnut skin test. He was also diagnosed with generalized anxiety disorder and Tourette’s syndrome at ages 7 and 10, respectively, and has had tics since age 5. He experienced diarrhea episodes nearly once a month and his skin was highly sensitive to touch. He also experienced reflux. Given his frequent hives, his primary physician assessed tryptase and prostaglandin D2 levels to assess for mast cell activation syndrome, however, both were within normal range.

His treatment history, per his allergist, included twice daily cetirizine (5 mg) and loratadine (10 mg) from age 3 to 6, then twice daily diphenhydramine from age 6 to 12. Despite this, approximately 1 month prior to referral for TCM, he presented to his allergist complaining of hives on most days. He was subsequently prescribed twice daily fexofenadine (180 mg), however, his hives persisted; they occurred almost every day, and sometimes several times per day. Although most reactions presented as hives, he also had severe reactions that required epinephrine, prednisone, and ER visits (Table 1).

**Fig.2:**
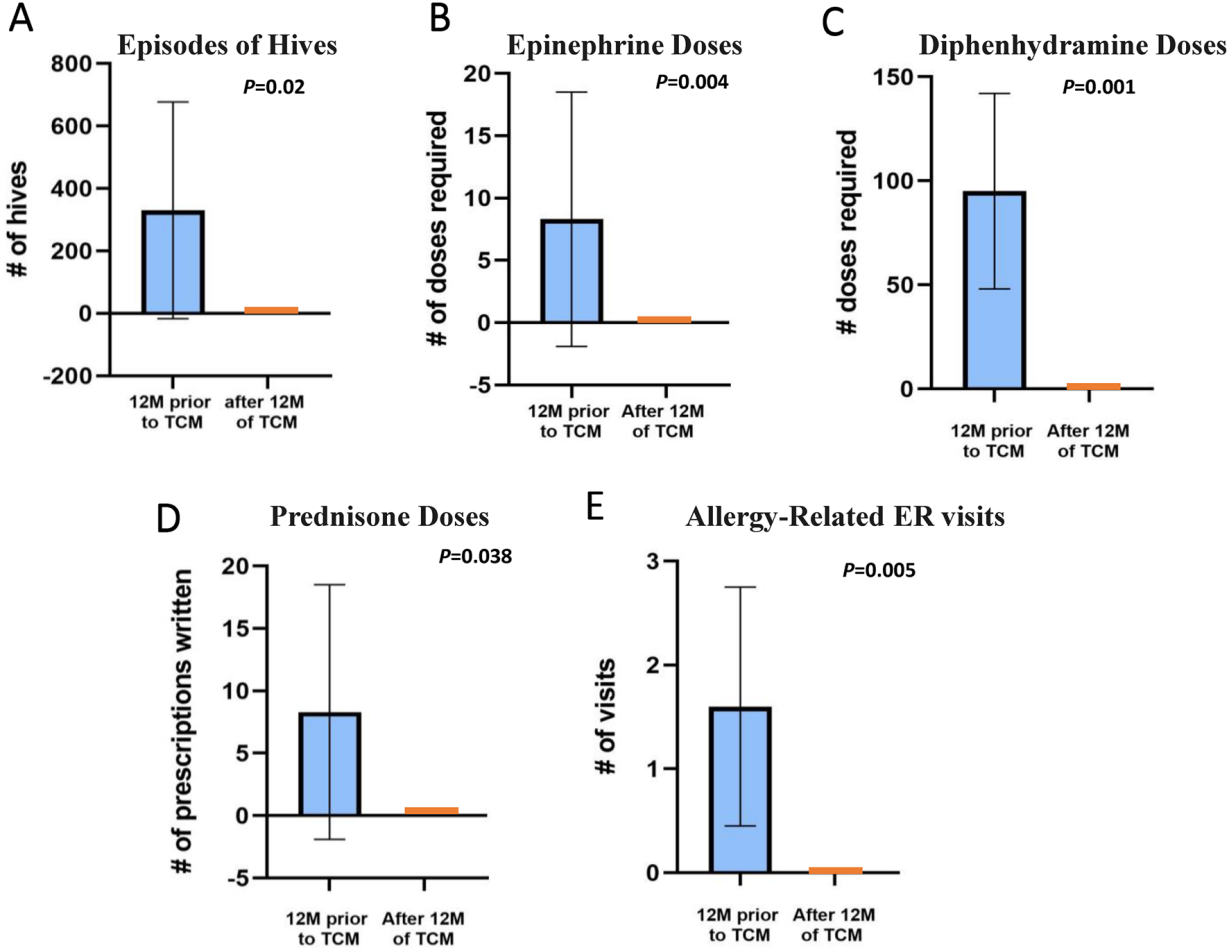
Decrease in incidence of atopic events with TCM therapy. “Before TCM” group reflects a mean total of all three patients, calculated 1 year prior to initiating TCM therapy. “After TCM” reflects the mean total events occurring 1 year after starting TCM

P2 began TCM therapy in October 2014 with the goal of reducing his allergic reactions and improving his general health. The TCM regimen included Remedy A-D with the addition of Good Mood tea (Remedy F) and Digestion Tea at 6 months. During 7–9 months of TCM treatment, P2’s hives episodes reduced to 0 (Table 1) and his food-specific IgE levels also started to decrease (Fig. 2). His diarrhea episodes, tics, and anxiety also improved. The goal of reducing allergic reactions, specifically hives, was achieved within 12 months of TCM therapy. After 2 years of TCM, he was tapered off of fexofenadine without recurrence of hives and started food challenges. By 3 years of TCM, he passed all challenges to previously offending foods. His allergist believes he no longer has food allergies, and P2 is now tapering off TCM therapy.

### Case 3

Patient 3 (P3) is a 20-month-old female with physician-diagnosed food sensitivity and allergies, chronic urticaria, and moderate-to-severe eczema. At 3 months of age, P3 was diagnosed with severe eczema by a dermatologist, and between 3 and 7 months of age, she began reacting to milk-based formula, soy-based formula, and elemental formula until, at 8 months of age, she was diagnosed with multiple food allergies. IgE analysis at this time revealed sensitization to dairy, wheat, soy, egg, peanut, and rye. She was put on daily desonide, yet extreme pruritus persisted. Her parents were not willing to step up to corticosteroid therapy given the side-effect profile at her age.

Most reactions were hives until 14 months of age when she experienced anaphylaxis from a veggie burger. She was treated with epinephrine in the ER and discharged home with prednisone and ranitidine. Subsequent testing revealed additional sensitivities to sesame seeds, as well as sunflower seeds, tree nuts, and many legumes. After the anaphylactic incident, she developed daily hives and was diagnosed with chronic urticaria. She concurrently developed severe environmental allergies, diarrhea up to eight times a day, and psychiatric complaints, including new-onset phobia of water.

P3 began TCM therapy in July 2015 to reduce hives and food reactions. Her basic regimen included Remedy A, B, and C, and additional Remedy Seasonal tea (Remedy G, 0.5 g, b.i.d) to reduce her seasonal nasal symptoms. Within nine days of initiating TCM Therapy, the eczema on her face and the thick, crusty cradle cap on her scalp were gone. Within 3 months of TCM, her episodes of hives were reduced from a daily occurrence to 1 episode during a 3-month period, for which she did not even use antihistamines. After 6 months of TCM therapy, she was completely hives-free. Her eczema, environmental allergies, chronic diarrhea, appetite and growth retardation all improved, as well. After 1 year of TCM, P3’s food specific IgE levels dropped to normal (Fig. 1), and she subsequently began introducing foods including vegetables, fruits and beans. She continues on TCM with the goal of introducing more foods to her diet. She is still hives-free.

## Discussion

This case series describes three patients with known histories of multiple food sensitivities and physician-diagnosed food allergies who also presented with severe, persistent, and difficult-to-treat hives. These three patients underwent TCM therapy with the goal of reducing the frequency and severity of their daily clinical symptoms associated with food allergy. After TCM therapy, these three patients experienced significant clinical improvement (Fig. 3), as evidenced by the decreased frequency and eventually elimination of skin reaction episodes, increased number of tolerated foods, improvement of other allergy-related conditions (eczema, asthma, environmental allergies), and improvement of general health (appetite, growth, mood). These patients also became less dependent on medical management and eventually discontinued all antihistamines and steroids.

Additionally, there were notable decreases in total IgE and allergen-specific IgE levels in response to both environmental and food antigens (Fig. 1). Although the food-specific IgE concentration does not necessarily correlate with clinical tolerance to the food, the fact food-specific IgE counts were up-trending prior to TCM, and rapidly reversed to steeply down-trending immediately following TCM, points to mechanistic interrelatedness. TCM is thought to tightly regulate mast-cell activation, so it is not surprising that climbing IgE levels were reversed and down-trending following TCM. Also, increased tolerance is accompanied by decreased IgE levels in OIT studies [22].

However, there are limitations to this report given the retrospective design, including recall bias, possible over-interpretation, and the fact that it was not a controlled study.

Although cases of comorbid food allergy and chronic urticaria are rare, this group of pediatric patients is in dire need of therapeutic options. Currently, there is a paucity of information available to support specific, novel treatment options for the management of pediatric patients with chronic urticaria. There are also no published cases of persistent and difficult-to-treat hives prior to this study.

In conclusion, this study highlights TCM therapy as a potential treatment option for children with multiple food sensitivities and chronic urticaria because all 3 cases demonstrated remarkable improvement following TCM. Future prospective clinical studies and investigation of the mechanism underlying the effect are needed.

## Supplementary Information

**Additional file 1.** Traditional Chinese medicine therapies for atopic conditions are comprised of numerous herbal components which interfere with the atopic inflammatory response, the mechanisms of which are well-described in the literature. The TCM formulations provided to these patients are included here with a breakdown of the herbal components of each.

## Data Availability

Not applicable.
