# Supplementary material for: Successful management of chronic urticaria and food allergies in a pediatric population using integrative traditional Chinese medicine therapy: a case series
Source: Clin Mol Allergy. 2022 Nov 25;20:12. doi: 10.1186/s12948-022-00175-y (PMC9700962; doi:10.1186/s12948-022-00175-y)
Supplement: Supplementary file 1 — Additional file 1. Traditional Chinese medicine therapies for atopic conditions are comprised of numerous herbal components which interfere with the atopic inflammatory response, the mechanisms of which are well-described in the literature. The TCM formulations provided to these patients are included here with a breakdown of the herbal components of each. [file 12948_2022_175_MOESM1_ESM.docx]

**Appendix A**

**Online Repository**

**Method: Herbal constituents:**

**Herbal Bath Additive** (*Phellodendron chinensis* formula, **Remedy A**) is dried

aqueous extract of *Cortex Phellodendri*, *Radix Rhizoma Rhei*, *Radix Sophorae Flavescentis,*

*Cortex Dictamni*, *Dayscarpi Radicis*, *Fructus Tribuli Terrestris*, and *Rhizoma Smilacis Glabrae*

in granule form (10g/pack per bath, once daily). **Herbal Cream III (***Phellodendron chinensis* topical cream, **Remedy B),** contains 2.8% *Phellodendron chinensis* and 1% *Indigo naturalis* extracts, use after daily bath. **Shi Zhen Tea Ia** (Burdock formula, **Remedy C)**) is dried aqueous extracts of *Herba Schizonepetae*, *Cicada Molting, Bombyx Batryticatus, Fructus Arctii Lappae, Rhizoma Atractylodis, Macrocephalae, Sophora flavescens Ait* in capsule form (0.5g/capsule, 5 pills b.i.d for P2, and 2 pills b.i.d for P3). **Mei Huang Tea** (*Pruni Mume* *formula* (**Remedy D***)* is an ethanol purified dried aqueous extract of *Prunus mume*, *Zanthoxylum schinifolium, Angelica sinensis*, *Zingiber officinalis*, *Cinnamomum cassia*, *Phellodendron chinensis*, *Panax ginseng*, and *Ganoderma lucidum* in capsule form (0.55g/capsule, 2 pills b.i.d for P1, and 5 pills b.i.d for P2); **Digestion tea** (*Fructus Jujubae Formula,* **Remedy E**) is *dried aqueous extract of Fructus Jujubae, Endothelium Corneum Gigeriae Galli, Fructus Amomi, Rhizoma Zingiberis Recens, Radix Pseudostellariae, Rhizoma Cyperi, and Semen Coicis* in capsule form (0.5g/capsule, 3 pills, BID for P1, 5 pills bid for P2 and 1 pill bid for P3). **Good Mood Tea (**Fructus Corni formula, **Remedy F)** is dried aqueous extracts of *Concha Ostreae, Sclerotium Poriae Cocos, Ramulus Uncariae cum Uncis, Fructus Corni, Acori Tatarinowii Rhizoma, Fructus Alpiniae Oxyphyllae, Fructus Jujubae, Radix Glycyrrhizae Preparata* (0.5g/capsule, 5 capsules b.i.d for P2). **Seasonal Tea (**Herba Centipdae formula, **Remedy G**) is dried extract of *Herba Centipdae, Fructus Luffae Retinervus, Fructus Arctii, Spica Prunellae, Flos Caryophylli*, 0.5g, bid for P3).

The remedies A, B were for external use. Remedies *C-F were* *internal use as dietary supplements.* Children who could not take capsules would take tea by dissolving the granules (extract) from the capsules/packs at the doses indicated into 15 mL of water or take the granules by mixing with soft foods such as applesauce. *All remedies, except remedy B,* were produced by Brion Herbs Corporation (Irvine, *CA).* Remedy B was produced by US Times Technology (Elmsford, NY). The tests for heavy metal, pesticide residual and microbial content met required standards.
